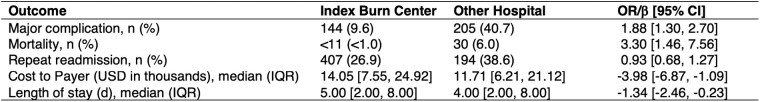# 588 Readmission to Non-burn Center Is Associated with Higher Complication and Mortality Rates in Burn Survivors

**DOI:** 10.1093/jbcr/iraf019.217

**Published:** 2025-04-01

**Authors:** Allen Green, Jeff Choi, Clifford Sheckter

**Affiliations:** Stanford University School of Medicine; Stanford University Department of Surgery; Santa Clara Valley Medical Center

## Abstract

**Introduction:**

Readmission to a non-index hospital has been associated with worse outcomes. Burn care is highly regionalized, and patients are often admitted to burn centers distant from their residence. After discharge, patients may seek emergent post-injury care at the closest hospital which is not the index burn center. We hypothesized that readmission to a non-index hospital would be associated with higher odds of major complications and mortality.

**Methods:**

We identified adults who experienced 90-day readmissions after index burn center hospitalization for burn injuries using data from the 2019 and 2020 National Readmissions Database. We defined readmissions as those that are reasonably attributable to injury sequelae and burn centers as hospitals with over 100 weighted burn admissions in a calendar year. Patients with index and non-index hospital readmission were matched based on age, sex, median household income, primary payer, total body surface area of burn (TBSA), initial operative management, initial disposition, and major diagnostic category using an optimal full matching algorithm with regression-based propensity score estimation. Weighted logistic and median regression models assessed outcomes associated with fragmented burn care. Outcomes included major complications (pneumonia, renal failure, sepsis, pulmonary embolism, cerebrovascular accident, myocardial infarction, cardiac arrest, respiratory arrest, and shock), subsequent readmission, mortality, length of stay, and cost to payer.

**Results:**

Among 19,422 weighted burn patients, 2,002 (10.3%) experienced 90-day readmission after discharge from initial hospitalization. 24% (N=490) were readmitted to a non-index hospital. Skin graft failure (5.7%) and septicemia (16.1%) were the most common primary reason for index and non-index readmission, respectively. Higher burn TBSA (OR [95%CI]: 0.11 [0.01,0.94]) and operative management during initial admission (OR [95%CI]: 0.62 [0.48,0.80]), were associated with lower odds of 90-day readmission to non-index hospitals. Compared with patients readmitted to their index hospital, those re-admitted to a non-index hospital had 88% increased odds of major complications (OR [95%CI]: 1.88 [1.30,2.70]) and 230% increased odds of mortality (OR [95%CI]: 3.30 [1.46,7.56]). Additionally, these patients had median readmission cost savings of $3980 (Median [95%CI]: -3.98 [-6.87,-1.09]) and their median length of stay was shorter by 1.34 days (Median [95%CI]: -1.34 [-2.46,-0.23]).

**Conclusions:**

One quarter of all burn patients readmitted were treated at facilities that were not the original burn center. Readmission to the non-index hospital was associated with an increased rate of major complications and mortalities.

**Applicability of Research to Practice:**

Burn centers should engineer systems to encourage their patients to be readmitted to their center when feasible. Readmission to other facilities is associated with worse outcomes.

**Funding for the Study:**

N/A